# Rare human papillomavirus 16 E6 variants reveal significant oncogenic potential

**DOI:** 10.1186/1476-4598-10-77

**Published:** 2011-06-24

**Authors:** Ingeborg Zehbe, Hava Lichtig, Ashley Westerback, Paul F Lambert, Massimo Tommasino, Levana Sherman

**Affiliations:** 1 Thunder Bay Regional Research Institute, Probe Development & Biomarker Exploration, Thunder Bay, Ontario, Canada; 2 Department of Clinical Microbiology and Immunology, Sackler School of Medicine, Tel-Aviv University, Tel-Aviv, Israel; 3 McArdle Laboratory for Cancer Research, University of Wisconsin, Madison, WI, USA; 4 International Agency for Research on Cancer, World Health Organization, Lyon, France

## Abstract

The aim of this study was to determine whether low prevalence human papillomavirus (HPV) 16 E6 variants differ from high prevalence types in their functional abilities. We evaluated functions relevant to carcinogenesis for the rarely-detected European variants R8Q, R10G and R48W as compared to the commonly detected L83V. Human immortalized keratinocytes (NIKS) stably transduced with the E6 variants were used in most functional assays. Low and high prevalence E6 variants displayed similar abilities in abrogation of growth arrest and inhibition of p53 elevation induced by actinomycin D. Differences were detected in the abilities to dysregulate stratification and differentiation of NIKS in organotypic raft cultures, modulate detachment induced apoptosis (anoikis) and hyperactivate Wnt signaling. No distinctive phenotype could be assigned to include all rare variants. Like L83V, raft cultures derived from variants R10G and R48W similarly induced hyperplasia and aberrantly expressed keratin 5 in the suprabasal compartment with significantly lower expression of keratin 10. Unlike L83V, both variants, and particularly R48W, induced increased levels of anoikis upon suspension in semisolid medium. R8Q induced a unique phenotype characterized by thin organotypic raft cultures, low expression of keratin 10, and high expression of keratins 5 and 14 throughout all raft layers. Interestingly, in a reporter based assay R8Q exhibited a higher ability to augment TCF/β-catenin transcription. The data suggests that differences in E6 variant prevalence in cervical carcinoma may not be related to the carcinogenic potential of the E6 protein.

## Background

Development of cervical cancer is strongly associated with the infection of high risk human papillomavirus (HPV), with HPV16 having the highest prevalence rate (50-70%) [1-4]. Cervical cancer is a rare consequence of high-risk HPV infection and additional viral and non viral factors are involved in determining the risk for cancer development. There are four key viral factor determinants that contribute to the progression of low grade lesions to invasive cancer: 1) persistence of HPV infection, 2) contentious expression of the viral oncogenes E6 and E7, 3) integration of viral DNA into the host cell chromosomes and 4) inactivation of the E2 gene. Furthermore, host factors such as HLA genotypes and polymorphism of p53 and methylenetetrahydrofolate reductase are also important determinants [4,5]. In fact, considerable evidence has been gathered on polymorphism in the HPV genome and risk for cancer progression.

Diversity arises within any given HPV genotype via limited nucleotide changes in the coding (< 2%) and non-coding (5%) regions [5]. These variants phylogenetically segregate based upon their geographical origin and thus are named European, African, Asian, Asian-American and North American. Significant differences in pathogenicity exist between variants within a single genotype and have been elucidated most clearly for HPV16. Multiple studies have demonstrated that HPV16 variants differ in their association with cervical cancer [6-15], viral persistence [16-21] and the frequency of recurrence of cervical disease [15]. Moreover, several studies investigated the functional consequence of changes in the viral LCR, E2 and E6 gene with respect to promoter activity, HPV gene expression and genome replication as well as cell transformation and related molecular pathways [4,5]. Most of the functional and mechanistic studies on carcinogenesis-related functions have been carried out on HPV16 E6 variants [22-27].

Previous investigations by our group and others indicated that E6 variants differ in their abilities to bind to the calcium-binding protein, E6BP, an E6 target involved in differentiation; to target bax, a pro-apoptotic protein for degradation [24], to inhibit serum/calcium-induced differentiation [22,25], to abrogate human keratinocyte phenotypic differentiation and expression of cytokeratins when grown in organotypic raft cultures [26] and to transform spontaneously immortalized aneuploid human keratinocytes [23] as well as foreskin primary human keratinocytes [27].

In a previous study we investigated the functional characteristics of L83V-related E6 variants that are highly represented in cervical cancers [7,8,14,19], including the Asian-American Q14H/H78Y/L83V variant [26]. The study found that variants differed from the E6 prototype in their abilities to dysregulate keratinocyte differentiation in organotypic cultures and modulate detachment-induced apoptosis (anoikis).

In the present study, we continued our investigation on the role of HPV E6 polymorphisms in the development of cervical cancer. We asked whether E6 variants with low prevalence in cervical carcinoma of European women have different carcinogenic potential than high prevalence variants. Three rare variants were characterized for their functional abilities in assays relevant to carcinogenesis when compared to a common variant in cervical cancer of European women, namely L83V. The rare variants contain a single amino acid variation in the N-terminus of E6. These include the R8Q, identified in one high-grade lesion of a Finnish woman [14], R10G, identified in two cervical carcinomas of Swedish women [12] and R48W identified in one cervical carcinoma of an Italian woman [12]. We provide evidence that low prevalence E6 variants possess significant, though different activities in the dysregulation of keratinocyte differentiation, modulation of suspension-induced apoptosis (anoikis) and augmentation of Wnt signaling. Thus, no distinctive phenotype that could be assigned to all rare variants could be identified.

## Materials and methods

### Human papillomavirus 16 E6 genotypes

We have chosen four HPV16 E6 variants, which had previously been detected in clinical samples from Swedish, Finnish and Italian women: the European R8Q, R48W, R10G and L83V [7,12,14]. The E6 genotypes tagged with HA at the carboxy-terminus were cloned into the LXSN vector and pJS55 vector as described previously [24,26].

### Transfection and retroviral gene transfer

High titer retroviral supernatants were generated by transient transfection of the Phoenix cell line (amphotropic virus). Infection of NIKS and selection in medium containing 150 μg/mL G418 were carried out as described [26]. Transfections into HEK293T and C33A cells were carried out with the jetPEI reagent (polyPlus transfection).

### Detection of HPV E6/variant expression

To verify the presence of DNA of each E6 variant in the recipient cells and to ensure E6 expression on the mRNA level, mRNA extracted from the transduced NIKS was reverse transcribed and amplified using the primers 5'- CAGCTGGGTTTCTCTACGTCT-3' and 5'-CATTTTCGTTCTCGTCATCTG-3' to detect the full length 16E6 transcript and the two splice variants. Protein expression was verified by Western blot analysis (anti E6 antibody and anti HA antibody), and immunofluorescence (anti HA antibody) as described previously [24,26]. The variation in R8Q hindered recognition by the anti HPV16 E6 clone 6F-4 [26].

### Western blot analysis

One hundred and twenty μg of protein lysate were loaded onto a 15% SDS-PAGE mini-gel, transferred to a PVDF membrane and immunoblotted. For detection of E6 variants, the monoclonal HPV16 E6 antibody (Euromedex, Strasbourg, France) [26] or anti HA rabbit polyclonal antibody (Y-11) (Santa-Cruz Biotechnology Inc.) were used at 1:1000. For detection of actin, goat anti-actin (sc-1616, Santa-Cruz Biotechnologies Inc.) was used at 1:1000. Detection of p53 was carried out using mouse anti-p53 (clone Do-7, DAKO, Mississsauga, ON, Canada) at 1:1000. A goat anti-mouse secondary antibody conjugated to horseradish peroxidase (Jackson Immunoresearch #115-036-071) was used either at 1:10.000 (for E6) or at 1:50.000. Detection of E6, p53 or β-actin was achieved by using the ECL Plus Western Detection Kit for horseradish peroxidase (Amersham, Piscataway, NJ, USA).

### Determination of p53 protein levels by ELISA

After treatment with 0.5 nM AD NIKS were harvested by trypsinization, counted and 1 × 10^5^cells/sample used per read-out. The cells were lysed and remaining levels of p53 were measured by Diaclone's ready-to-use fully formatted ELISA kit (Diaclone, Besancon, France). The mean value/STD of 3 independent experiments using duplicates for each cell line was calculated with MS Excel.

### Organotypic raft cultures and immunofluorescence

Raft cultures derived from NIKS stably transduced with LXSN and HPV16 E6 variants were grown in triplicate for 14 days and processed for immunofluorescence as previously described [26]. Immunofluorescence on raft culture sections (5 μ) was performed using the appropriate primary antibodies: a rabbit anti-K5 polyclonal antibody (#24647, Abcam, Cambridge, MA, USA), a mouse anti-K10 monoclonal antibody (DAKO, Mississauga, ON, Canada), or an anti human K14 antibody (MLA890T, SeroTec), followed by secondary antibodies, AlexaFluor 488 donkey anti-rabbit (Invitrogen) or AlexaFluor 594 donkey anti-mouse (Invitrogen), as described [26,27]. A Nikon eclipse 80 i microscope configured with a Nikon digital camera Dxm 1200 c was used to image the fluorescent-stained sections with 350, 488 and 594 lines for blue, green and red channel excitation, respectively. Exposure times were adjusted to each colour channel, identical for each respective image. All colour images were saved in a Tag Image File Format (TIFF) and were used for quantitative fluorescence analysis.

### Quantitative fluorescence analyses

The digital microscopy images were quantified and the average fluorescence intensity of each region of interest was calculated using the Multi Image Quantification Analysis System; Cytoview, Petach Tikva, Israel as described [26,28]. Four images of each staining were captured from different sections and used for quantitative analyses. The regions of interest, basal and suprabasal, were defined manually in each image, according to the tissue morphology of the DAPI stain. Finally in order to compare the relative levels of protein expression in each region, the average area intensity values were used.

### Induction of apoptosis in semisolid medium

To prepare semisolid medium 3.37 g of methylcellulose was autoclaved with a magnetic stir bar in a 250 ml bottle. 100 ml of serum-free medium (Ham's F12 and DMEM at a ratio of 3:1 as well as Ca^2+ ^at a final concentration of 0.66 mM) was heated to 60°C, added to methylcellulose, stirred for 20 min at RT followed by adding an additional 100 ml medium and stirred at 4°C for 1 h. The semisolid medium was then centrifuged for 90 min at 10,000 rpm in 50 ml Falcon tubes to remove non-dissolved methylcellulose fibers. NIKS were trypsinized, washed once with serum-containing NIKS medium followed by one wash with serum-free medium. NIKS were suspended in semisolid medium at a density of 1 × 10^6 ^cells/ml at 37°C and 5% C02 atmosphere for 16 h. Negative controls were adherent cells overlaid with semisolid medium. After incubation in semisolid medium, cells were washed three times with PBS, trypsinized, washed once in serum-containing medium and twice in PBS. Pellets were used for the Annexin V-FITC assay (Sigma) measured by flow cytometry.

### Luciferase reporter activity assay

Assays for induction of Wnt signaling and its augmentation by E6 variant expression were carried out as previously described [29]. HEK293T or C33A cells growing in 5 cm dishes were transfected at 60-70% confluence with DNA of the superTOPFLASH or the control superFOPFLASH (containing mutated TCF/β-catenin binding sites) (1 μg) plasmid, the β-gal (0.1 μg) expression plasmid (used to evaluate the transfection efficiency in HEK293T cells) or 0.5 μg Renilla luciferase (used to evaluate the transfection efficiency in C33A cells) and various amounts of the E6 variant plasmid. Wnt signaling was induced by cotransfection of the plasmids encoding the Wnt ligand, pWnt3a (0.1 μg), and the Wnt receptor, human Frizzeled 1, pHFz1 (0.3 μg). Empty vector DNA was added to adjust for equal amounts of transfected DNA. The jetPEI reagent, (polyPlus Transfection) was used for transfection. Forty eight hours after transfection, luciferase levels were measured using the Luciferase Assay System kit (Promega). The firefly luciferase activity was normalized relative to the β-galactosidase or the Renilla luciferase activity. Luciferase readouts obtained with the superFOPFLASH (0.01-0.001 of the superTOPFLASH signal) were subtracted from the readouts obtained with the superTOPFLASH plasmid. Data is presented as relative luciferase readouts (fold activation) where luciferase readouts in the vector cells were presented as 1. The collated results of fold activation are mean values and standard deviations (SD) from at least three independent experiments done in duplicates.

### Statistical analyses

Data of activity in the various functional assays is presented in the figures as average (mean) values + standard deviation (SD). Data was subjected to one-way ANOVA. Significance was accepted at *P *< 0.05.

## Results

**Figure 1:**
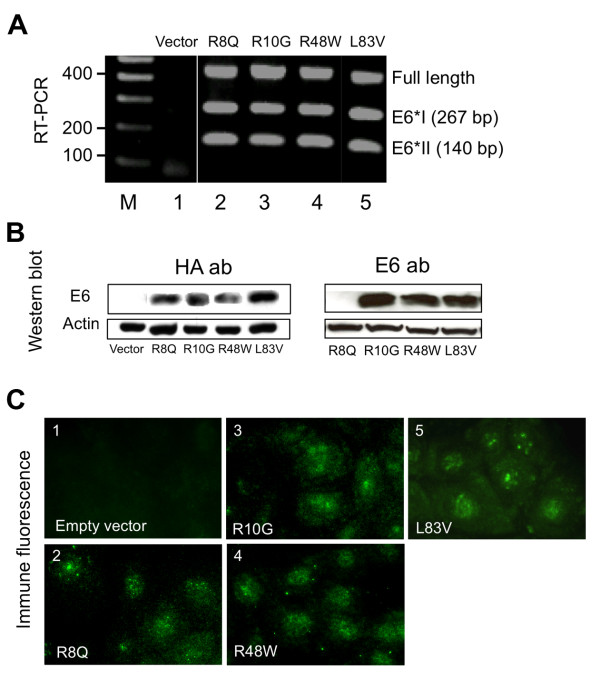
**Gene expression of HPV16 E6 variants in NIKS as defined by reverse transcriptase polymerase chain reaction (RT-PCR), Western blot and immunofluorescence**. RT-PCR detected three bands representing mRNAs of the full length and the splice variants E6I and E6II (A). With the E6 antibody used (clone 6F4) and input of 120 μg of total protein, a band of the expected size was detected in extracts of NIKS transduced with all E6 proteins except R8Q. Loading of 200 μg total protein detected a faint band (data not shown). Western with the anti-HA antibody detected all the E6 proteins (B). The micrograph shows immunofluorescence against the HA-tag epitope. E6-specific staining is present in all transduced NIKS lines (C).

In this study, we evaluated the functional abilities of rare HPV16 E6 variants, i.e. those with low prevalence in cervical carcinomas of previously studied European populations, including variant R8Q, R10G, and R48W [12,14]. These variants were tested for activities considered to be important in HPV associated carcinogenesis. The E6 L83V, which is highly represented in European populations and in cervical carcinomas, was included for comparison. The E6 mRNA and protein expression of the variants was verified. Results are summarized in Figure 1.

### E6 polymorphisms similarly abrogate growth arrest and inhibit elevation of p53 induced by actinomycin D

**Figure 2:**
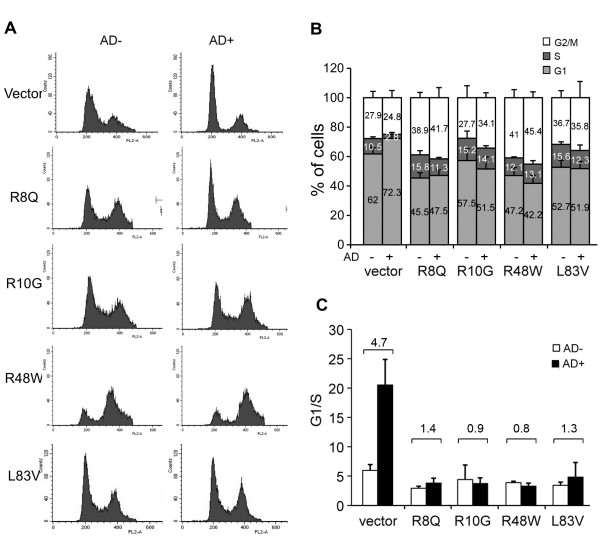
**Cell cycle profile of HPV16 E6 variants**. The distribution of cells in G1, S and G2/M phases, obtained by flow cytometry, is demonstrated in A (histograms from a representative experiment) and B (mean values bars). Data with and without actinomycin D (AD) treatment (0.5 nM for 24 h) are shown. Differences in G1/S ratio are depicted in C. Mean values of at least three independent experiments are presented as average (mean) ± standard deviation (SD). Significance was tested by a one-way ANOVA test. A *P *value of < 0.05 was considered significant.

**Figure 3:**
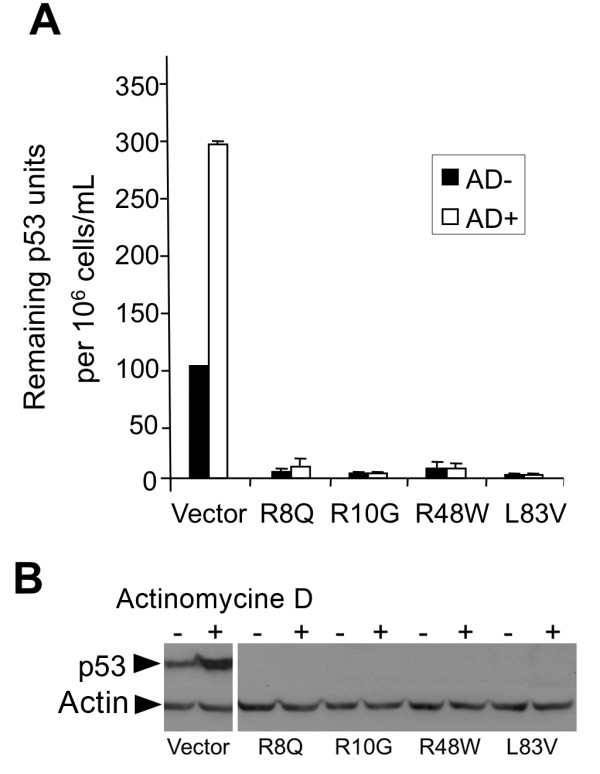
**p53 level in HPV16 E6 variants NIKS before and after treatment with actinomycin D**. (A) ELISA showing the remaining units of p53 quantified according to the number of cells after AD treatment (0.5 nM for 24 h). Mean values of at least three independent experiments are presented as average (mean) values ± standard deviation (SD). Significance was tested by a one-way ANOVA test. A *P *value of < 0.05 was considered significant. (B) The corresponding Western blot showed only p53-specific bands for the empty vector NIKS. After AD treatment the amount of p53 was increased approximately threefold as seen with ELISA.

The ability of high risk HPV E6 to inhibit growth arrest and to abrogate DNA damage responses induced by p53 is crucial during HPV-associated carcinogenesis [30]. The capacity of the E6 protein to abrogate actinomycin D (AD)-induced growth arrest was correlated to its ability to target p53 for degradation *in vivo *[31]. We examined the ability of the E6 variants to override growth arrest and to reduce p53 levels induced by AD. The distribution of cells in the G1, S and G2/M phases and the G1/S ratios were determined before and after the treatment with AD (Figure 2A-B). Treatment of the control vector NIKS resulted in a dramatic increase of growth-arrested cells, as evidenced by the reduction in the proportion of cells in S phase and increase in the proportion of cells in G1 phase with an increase in the G1/S ratio (Figure 2C). In contrast, no significant change in the G1/S ratio was observed in E6-transduced NIKS, indicating that all E6 variants alike could overcome the growth arrest induced by AD (Figure 2C). In the empty vector control cells, the ratio increased about 5-fold, while it stayed close to 1 (range 0.8 to 1.4) in the E6-transduced NIKS in the presence of AD (Figure 2C). The highest efficiency in overcoming the G1 block was exhibited by the E6 variants R10G (r = 0.9) and R48W (r = 0.8), while R8Q showed the lowest efficiency (r = 1.4) (Figure 2C). Additionally, all variants demonstrated an increased proportion of cells in G2/M after AD treatment. In both cases, the inter-variant differences were not statistically significant. Consistent with these results, when compared to control NIKS all variants significantly lowered the steady-state levels of p53 and were able to prevent the elevation of p53 after treatment with 0.5 nM AD for 24 h. However, the p53 level increased about 3-fold in the vector control NIKS after treatment, as evidenced by ELISA and Western blot (Figure 3). These results showed no statistically significant difference in the activities between the rare (R8Q, R10G, R48W) and common L83V variants, consistent with previously described data [24,26].

### E6 polymorphisms selectively alter keratinocyte differentiation induced in organotypic keratinocyte raft cultures

Squamous epithelia consist of proliferating, basal, and differentiating suprabasal layers of keratinocytes. Keratinocyte stratification and differentiation is a tightly regulated process [32,33]. The epidermal layers are characterized by intermediate filament structures denoted keratins. While keratin pairs K5 and K14 are expressed in the basal layer, K1 and K10 are expressed in suprabasal layers starting with the spinous layer. Due to the fact we found aberrant morphology and differentiation patterns (phenotypes) in keratinocyte cultures transduced with the high prevalence variants [26] we determined it necessary to investigate the pattern of differentiation induced by low prevalence variants.

**Figure 4:**
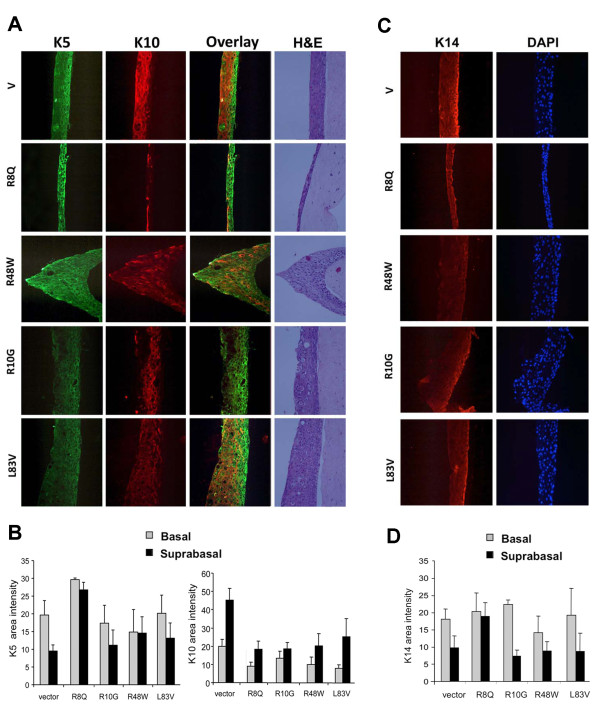
**Keratinocyte differentiation induced in 3D raft cultures**. (A) The panel depicts 3D raft cultures obtained from NIKS transduced with the indicated HPV16 E6 variants and vector. Micrographs include immunoftuorescence for the basal cell marker K5 (green), suprabasal cell marker K10 (red), the overlay of both and H+E staining. Rafts had been grown three times and sections were cut twice from each set of paraffin blocks to ensure reproducibility. (B) Calculation of K5 and K10 levels are shown for each HPV16 genotype and the empty vector raft as described in Materials and Methods. Values of area intensities for K5 and K10 in the basal and suprabasal regions from 4 sections are presented as mean ± standard deviation (SD). (C) Raft cultures were produced as described in (A). Micrographs showing immunoftuorescence of the basal cell marker K14 (Red) and nuclear staining of the same section (DAPI; blue) are shown. (D) Calculation of K14 levels in the basal and suprabasal regions were carried out as described in (B). Significance was tested by one way ANOVA test. A *P *value of < 0.05 was considered significant.

To evaluate the abilities of the HPV16 E6 variants to alter phenotypic differentiation, we grew the E6-transduced NIKS in organotypic raft cultures and looked for alterations in the three dimensional structure and expression of specific keratins. NIKS underwent a normal differentiation program, comparable to normal skin when cultivated in organotypic raft cultures [34]. Haematoxylin and eosin staining of raft cultures transduced with the empty vector showed ordered stratification and epithelial thickness as previously described for normal NIKS [34]. A layer of undifferentiated basal cells, several layers of suprabasal cells and anucleated cells from the cornified envelope could be detected (Figure 4A). Raft cultures obtained from NIKS transduced with the E6 variants were different in morphological appearance. All cultures were dysplastic with abnormal stratification, showing more or less than the expected epithelial thickness, nuclear atypia and lack of cell polarity (most pronounced in the R48W raft). No cornified layers (anucleated cells) were seen suggesting lack of terminal differentiation. Nuclei were present in all layers; however mitotic figures, usually observed in epithelial dysplasia, were absent (Figure 4A). The raft cultures derived from R8Q and R48W demonstrated mostly undifferentiated cells with little cytoplasm, while L83V and R10G demonstrated strata with undifferentiated and differentiated keratinocytes. The R8Q variant raft showed a considerably lower number of epithelial layers than the raft cultures expressing the vector LXSN. Other E6 variants showed increased thickness compared to LXSN characteristic of hyperplasia.

To examine whether keratin marker expression varied amongst E6 variant raft cultures, we performed immunofluorescence using antibodies against both the basal cell markers K5 and keratin 14 and the suprabasal cell differentiation marker K10 (Figure 4A-D). In the vector control NIKS, basal cell type markers K5 and K14 were expressed in the basal cell compartment and K10 was expressed in the suprabasal cells as expected. Thus, control rafts demonstrated a complementary staining pattern of differentiation markers similar to normal skin. Rafts expressing the E6 proteins exhibited perturbed epithelial differentiation. K5 was expressed in basal and suprabasal layers independent of the E6 subtype, suggesting a phenotype of early sqamous cell carcinoma for both rare and common variants [35,36]. The area intensity of K5 in the suprabasal layers was the strongest in the R8Q raft culture with the difference in intensity from LXSN, L83V, R10G and R48W being statistically significant (Figure 4A-B). K10 staining was mainly detected in the suprabasal cell layers. Staining was uniform in the L83V rafts, whereas only sporadic staining was observed in the R8Q, R10G and R48W rafts (Figure 4A-B). R8Q exhibited K10 staining in the uppermost layer only. The area intensity of K10 in the suprabasal layers of all E6 variant rafts was significantly lower than that of the vector. K14 another marker of basal cells, was mainly expressed in the basal layer of rafts, except for R8Q, which showed uniform staining in all layers (Figure 4C-D). The area intensity of K14 in the suprabasal part of the R8Q rafts was significantly higher than that of LXSN and all other E6 rafts.

### The ability of rare and common HPV16 E6 variants to modulate apoptosis induced by cell suspension in semisolid medium differs

**Figure 5:**
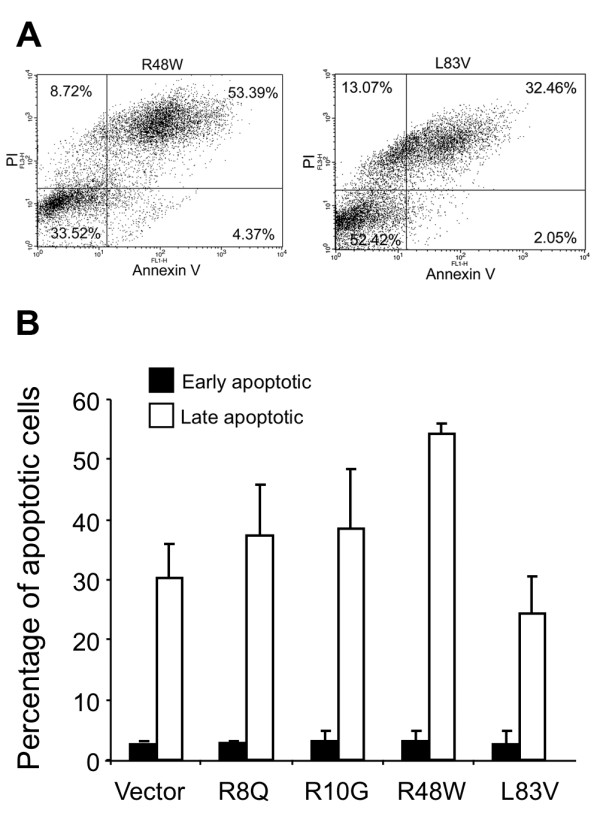
**Modulation of apoptosis by HPV16 E6 variants**. Flow cytometry was performed after a 16 h treatment in semisolid medium and apoptosis-alive (Annexin V-FITC+/propidium iodide-), apoptosis-dead (Annexin V-FITC+/propidium iodide+), intact-dead (Annexin V-FITC-/propidium iodide+) and intact-alive (Annexin V-FITC-/propidium iodide-) cells were calculated for each quadrant. (A) dot plots from a representative experiment. (B) Mean values of at least three independent experiments are presented as average (mean) values ± standard deviation (SD). Significance was tested by a one-way ANOVA test. A *P *value of < 0.05 was considered significant.

NIKS are permissive for cell-detachment-based apoptosis (anoikis), which is triggered by the extrinsic death receptor pathway [37]. Anoikis can be induced by suspending cells in a semisolid medium. NIKS were exposed to serum-free medium made semisolid with methylcellulose. NIKS kept in suspension underwent apoptosis as determined by annexin V-FITC labeling and FACS analyses (Figure 5A-B). A significant increase in the proportion of cells that stained with annexin V-FITC, characteristic to apoptosis, was observed in the vector control and E6-transduced cultures. A portion of these cells (24.3-54.5%) also stained with propidium iodide (annexinV+/PI+) indicating late apoptotic cells. A smaller fraction (2.4-5.6%) consisted of early apoptotic cells (annexinV+/PI-) with no significant difference in their proportion among the tested cultures. The L83V NIKS showed approximately 20% lower levels of apoptotic cells than LXSN NIKS. Conversely, the other E6 variant NIKS showed higher levels of apoptosis than the vector cells, indicating that the rare variants sensitize NIKS for suspension-induced apoptosis (Figure 5B). NIKS transduced with R48W exhibited the highest levels of late apoptotic cells, with the difference from the empty vector NIKS and NIKS expressing L83V being statistically significant (Figure 5B).

### HPV16 E6 variants show different abilities in the hyperactivation of Wnt signaling

**Figure 6:**
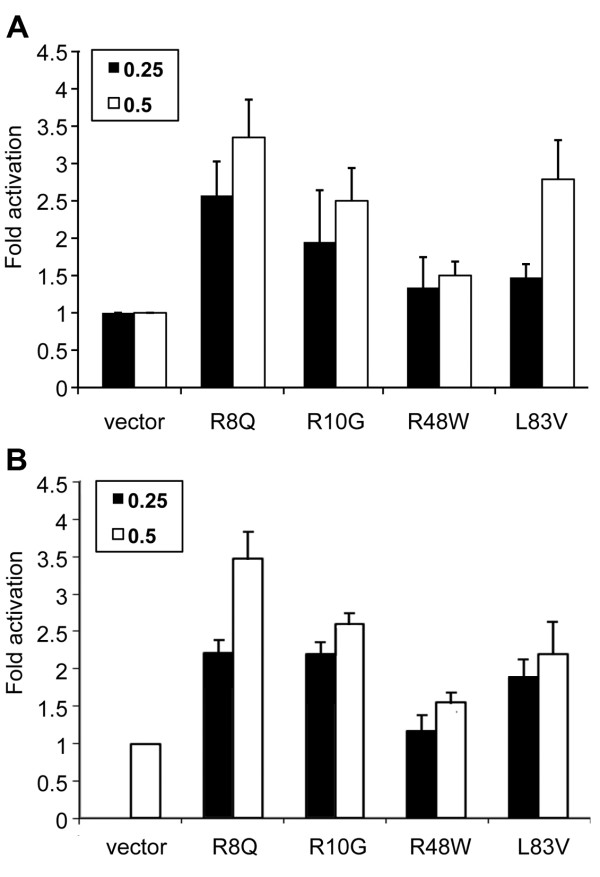
**Hyperactivation of β-catenin/TCF-dependent transcription induced by Wnt-3a and HFz1 in the context of E6 variants**. (A) HEK293T cells were co-transfected with TCF/LUC reporter (1 μg), β-Gal (0.1 μg), Hfz1 (0.3 μg), Wnt-3a (0.1 μg) and E6 variants as indicated. Each E6/variant was transfected at 2 amounts, 0.25, 0.5 μg plasmid DNA. (B) C33A cells were transfected as in indicated in A except that 0.5 Renilla luciferase DNA was transfected instead of β-Gal. Luciferase activities were measured 48 h following transfection. The histograms show fold activation relative to the control cells transfected with the vector DNA (pJS55). The average values of fold activation ± SD of the indicated plasmids are shown. Data are from at least 3 independent experiments with HEK293T and two experiments with C33A cells. Significance was tested by a one-way ANOVA test. A *P *value of < 0.05 was considered significant.

The role of the Wnt TCF signaling pathway in the development of cervical cancer has not been elucidated, although several reports have provided evidence on deregulation of this pathway in cervical cancers [38,39]. Previous studies showed that activation of the canonical Wnt signal in HPV immortalized keratinocytes induced their malignant transformation, while Wnt signal activation in cells absent of HPV did not [38]. Our recent studies demonstrated that the high risk HPV E6 augments canonical Wnt-TCF signaling [29]. In the present study, we evaluated the abilities of the E6 variants to hyperactivate Wnt signaling in a β-catenin/TCF reporter assay. Transfection of the Wnt3a ligand and HFz-1 receptor into HEK293T cells upregulated lucerifase activity. Cotransfection of E6 variant plasmids further augmented the Wnt/HFz-1-induced signal in a dose-dependent manner reaching up to 4-fold activation over that of the vector LXSN (Figure 6A). Consistent with our previous report, none of the E6 proteins were capable of inducing the luciferase reporter activity when transfected alone (data not shown). The E6 variants showed different abilities to augment Wnt signaling. The R8Q variant showed the highest hyperactivation activity and the R48W showed the lowest activity. The differences in activity between R48W and both R8Q and L83V were statistically significant. R10G and L83V variants also showed lower activity than R8Q but the difference was not statistically significant. Luciferase reporter assays carried out with C33A cells, a cervical carcinoma cell line that lacks HPV, showed similar results (Figure 6B). Although luciferase readouts in extracts of C33A cells were lower than HEK293T cells, signal augmentation by the different variants showed a comparable trend as that observed in HEK293T cells (Figure 6B).

## Discussion

Previous data suggests that variants of the same HPV type are biologically distinct and may confer different pathological risks [5]. We have previously found differences in the functional activities of common HPV16 E6 variants and sought to investigate how rare variants differed in these activities. Three variants with low presentation in cervical carcinomas of previously studied European populations, accounting for 0-2.2% in different cohorts [12-14], were investigated. Activities of these variants were compared with the L83V variant, which is highly represented in cancer of European populations. In this study, several activities considered to be important in HPV associated carcinogenesis were evaluated. The disruption of p53-mediated responses to DNA damage by E6 is believed to contribute to subsequent accumulation of genetic changes associated with cervical carcinogenesis [40]. Previous studies with site directed mutants demonstrated that other amino acid changes in the same positions as those of R8Q and R10G, did impair the ability of E6 to induce p53 degradation [31]. However, consistent with our earlier investigations [24-26] we show that all tested E6 polymorphisms were capable of strongly reducing the steady-state levels of p53 in NIKS.

This research also demonstrates that similar to L83V, the rare E6 variant proteins were able to both suppress the elevation in levels of the p53 protein and override p53-mediated growth-arrest that normally is induced by actinomycin D [30]. The similarities in the modulation of DNA damage responses between the common L83V and the rare E6 variants, both in terms of suppression of p53 accumulation and overcoming growth arrest, strongly suggest that these functions of E6 cannot be compromised to initiate the carcinogenic process.

Growth of HPV-immortalized cell lines in organotypic cultures produces epithelial layers exhibiting abnormal morphology with phenotypes similar to that observed in premalignant lesions and carcinomas found in vivo [27,41,42]. Expression of the E6 protein alone was also shown to induce alterations of keratinocyte differentiation in organotypic cultures, including disorganized layer formation and hyperplasia [[26] and references therein]. In a recent study, L83V-related variants of HPV 16 E6 were evaluated for alteration of NIKS differentiation, it was demonstrated that all tested E6 proteins induced hyperplastic cultures with abnormal stratification. In addition, deregulated expression of squamous cell differentiation proteins was detected in all E6-expressing rafts [26]. We show herein that the rare E6 variants also induce dysregulated differentiation of NIKS when grown in organotypic cultures, but with various phenotypes. R10G and R48W induced hyperplastic proliferation similar to that induced by the high prevalence type L83V, whereas the R8Q cultures had a unique phenotype, characterized by lack of stratification. Furthermore, the same variant was previously demonstrated to have high activity in the inhibition of serum- and calcium triggered stratification and terminal differentiation [25].

Perturbed differentiation in all E6 cultures was indicated by aberrant expression of K5 in suprabasal cells. Expression of K14 was more confined to the basal cells except for R8Q, which showed strong expression of both K5 and K14 throughout the rafts suggesting that the cells do have a basal cell phenotype. Although expression of K10 was restricted to the suprabasal layers of the raft cultures, a significantly lower expression was detected in the E6 NIKS with the rare variants R10G, R48W and R8Q. Interestingly, these E6 variants showed lower capacity to bind E6BP, a calcium binding protein involved in keratinocytes differentiation, as demonstrated in a previous study [24]. Simultaneous expression of K10 and K5 was also observed in the E6 cultures. This was more pronounced in the L83V and the R48W cultures. Replication of suprabasal keratinocytes aberrantly co-expressing K5 and K10 due to a premature migration of proliferating keratinocytes into the suprabasal compartment is a hallmark of early squamous cell carcinogenesis [35,43]. This may reflect an advantage for the variants exhibiting this characteristic in promoting carcinogenesis. On the other hand, the observed phenotype may impact on the viral life cycle, which is tightly linked to the differentiation program of the cell and typically occurs during early carcinogenesis. In this respect, strong activity in suppression of keratinocyte differentiation as that exhibited by R8Q may not be favorable for viral persistence, which may explain in part the rare detection of the R8Q variant in previously studied populations [12,14].

Suspension of normal keratinocytes into semisolid medium induces their terminal differentiation [44]. This trigger also induces detachment associated apoptosis (anoikis) in NIKS [34]. NIKS transduced with the minor E6 variants, R8Q, R10G and particularly R48W, exhibited higher apoptotic figures when suspended in low calcium and serum-free semisolid medium, indicating their ability to augment anoikis. The largest increase was in R48W and was statistically significant when compared to the vector cells. In an earlier investigation [26] NIKS transduced with the E6 variants Q14H/H78Y/L83V and R10G/L83V demonstrated significantly higher levels of late apoptotic cells when suspended in methylcellulose than L83V. The biological significance of this finding was suggested to be related to the means of viral transmission and spread in the tissue. Further confirmation of this comes from the fact that caspase-3 has been found elevated during the productive stage of the viral life cycle that occurs in differentiating keratinocytes [45]. High activity of the Asian American variant Q14H/H78Y/L83V in apoptosis induction [26] was suggested to be related to its high persistence and increased risk of progression to cancer (reported to be 20-fold higher than that of prototype E6) [13]. However, data obtained in this study suggests that, induction of high levels of apoptosis may not be a major determinant of viral persistence. As our data shows R48W with significantly higher ability to induce apoptosis than L83V, has low prevalence in previously studied European populations (identified in only one ICC of an Italian women) [12,14]. It appears that the differentiation ability as reflected by K10 expression is more important as it was found to be significantly higher among common compared to rare variants detected in cervical carcinomas [26,27]. Thus, the viral life cycle is largely dependent on the differentiation program of the cell.

Deregulation of the canonical Wnt/β-catenin signaling pathway often leads to the formation of epithelial tumors [46,47]. It was recently shown that the Wnt canonical signal controls the maintenance of skin cancer stem cells [48]. The role of the Wnt-TCF signaling pathway in the development of cervical cancer has not been elucidated, although evidence for deregulating this pathway in cervical cancers exists [38,39]. Experimentally, Wnt signaling activation in HPV immortalized keratinocytes induced their malignant transformation [38]. In a recent study, we demonstrated that HPV16 E6 augments Wnt/β-catenin signaling, which could be a way by which HPV oncoproteins may contribute to promoting transformation of human keratinocytes [29]. Luciferase reporter assays described herein indicated that the HPV16 E6 variant proteins were capable to hyperactivate TCF-β-catenin-dependent transcription. The rare variant R8Q exhibited significantly higher ability to augment Wnt/TCF-β-catenin transcription, an activity that could be of advantage in keratinocyte transformation. Interestingly, as indicated above, this variant inhibited stratification and differentiation of human keratinocytes; an event associated with the initial stages of keratinocytes transformation. Additional studies are needed to determine how enhancement of the canonical Wnt pathway by E6 contributes to transformation of human keratinocytes.

The underlying mechanisms for the differential phenotypes triggered by the various E6 proteins with respect to differentiation, anoikis and Wnt signaling augmentation remain to be determined. Differential binding to and altered expression of E6 targets involved in apoptosis [49] keratinocyte differentiation [32,33] and Wnt signaling [46,47,50] could be the reason for the observations described here.

## Conclusion

The aim of this study was to discover whether low prevalence E6 variants differ from high prevalence types in their oncogenic abilities. We found that rare variants similar to common variants exhibit significant activities in various assays relevant to cervical carcinogenesis. Based on our data in this and a previous study [26] several differences between the high and low prevalence types in biological activities were identified, but none of the activities were exclusively distinctive for either group. The common types may differ from the rare types in other biological functions relevant to transformation and development of cancer that were not tested in this study [4,5]. However, it is also possible that other determinants influence the prevalence of distinct viral variants in cancer, such as rates of transmission and persistence in certain populations (which depends on host genetic factors and immunogenicity) [4,5,20]. Indeed, both HLA type and E6 variation were shown to influence immune recognition by T lymphocytes, thereby impacting on prevalence of E6 variants in cervical cancer [12,51,52]. Thus both genetic factors in populations and functional activities of E6 variants likely contribute to cervical carcinogenesis. Based on the current data, low prevalence of distinct E6 variants in cervical cancer may not necessarily correlate with their carcinogenic potential. This data is important for the understanding of the molecular pathogenesis of HPVs and also have clinical implications for the management of HPV infected patients and future vaccine design.

## Competing interests

The authors declare that they have no competing interests.

## Authors' contributions

IZ participated in the design and interpretation of the study, carried out the majority of the experimental work, helped to draft the manuscript and critically revised it. HL helped in the design and interpretation of the data, carried out part of the experimental work and performed the quantitative fluorescence analyses. AW carried out part of the experimental work and helped with interpretation of the data. PFL and MT participated in the conception and design of the study and approved the final manuscript. LS conceived and designed the study, participated in part of the experimental work, and critically reviewed and communicated the manuscript. All authors have read and approved the final manuscript.
